# The Generation of a Nanobody-Based ELISA for Human Microsomal Epoxide Hydrolase

**DOI:** 10.3390/ijms241914698

**Published:** 2023-09-28

**Authors:** Qiyi He, Mark R. McCoy, Meng Qi, Christophe Morisseau, Huiyi Yang, Chengpeng Xu, Rachel Shey, Michael C. Goodman, Suqing Zhao, Bruce D. Hammock

**Affiliations:** 1 Department of Entomology and Nematology and University of California Davis Comprehensive Cancer Center, University of California Davis, Davis, CA 95616, USA; qiyhe@ucdavis.edu (Q.H.); mrmccoy@ucdavis.edu (M.R.M.); hpqm08@163.com (M.Q.); chmorisseau@ucdavis.edu (C.M.); iyiuhgnay@163.com (H.Y.); chpxu@ucdavis.edu (C.X.); rshey@ucdavis.edu (R.S.); michael.c.goodman@vanderbilt.edu (M.C.G.); 2 Department of Pharmaceutical Engineering, School of Biomedical and Pharmaceutical Sciences, Guangdong University of Technology, Guangzhou 510006, China

**Keywords:** nanobody, microsomal epoxide hydrolase, ELISA, biomarker

## Abstract

A microsomal epoxide hydrolase (mEH) metabolizes in vivo in both xenobiotic and endogenous epoxides associated with signaling function. Findings in patients suggest that mEH might be a biomarker for several diseases, including metastatic cancer and viral hepatitis. To easily quantify mEH, nanobodies specific to the human mEH were isolated from a phage library of llama VHHs. Four unique clones were obtained and used for developing ELISAs. Three formats of double antibody sandwich assays were investigated using different detection strategies. Using PolyHRP, the signal was strongly amplified, yielding a 22-fold lower LOD (12 pg mL^−1^) than the ‘conventional’. To further validate the performance of the immunoassays, human tissue samples were analyzed by nanobody-based ELISAs and compared to the enzyme activities (*R*^2^ > 0.95). The results demonstrate that these nanobodies are powerful tools for the quantification of human mEH and could eventually result in a bedside assay.

## 1. Introduction

The microsomal epoxide hydrolase (mEH, EPHX1, E.C. 3.3.2.9) was the first mammalian epoxide hydrolase discovered. It notably catalyzes the conversion of epoxides formed during the phase I metabolism of xenobiotics to vicinal diols [1]. While mEH has been historically studied for its role in the metabolism and toxicity of xenobiotics [2,3], patient data have indicated the endogenous biological roles of this protein over the years. In several human genetic studies, the expression of the high activity mEH mutant is associated with the development of several diseases, including cancer, preeclampsia, and neurological disorders [4,5,6,7]. Interestingly, on the opposite end, the absence of mEH activity results in a patient suffering from lipoatrophic diabetes syndrome and increased cellular senescence [8]. Although studies of the role of mEH in diseases are relatively sparse, it has been suggested that mEH is involved in the regulation of metabolism, and this would then be natural epoxy-fatty acids (EpFA) [9]. EpFAs are involved in regulating many biological functions in the organism, leading to diseases when their levels are unbalanced [10]. The role of mEH in EpFA homeostasis appeared complementary to soluble epoxide hydrolase (sEH) [11,12], and thus mEH could be a potential therapeutic target [13]. Therefore, monitoring mEH levels in tissues and body fluids may be useful for epidemiology association studies to mEH and could provide a powerful diagnostic tool for pharmacological studies.

While mEH is mainly found in the endoplasmic reticulum (ER) of cells pertaining to several organs, especially the liver [1], mEH becomes pseudo-soluble under pathological conditions and can be detected in the plasma of a patient. In the 1980s, a novel protein was found to dissociate from the endoplasmic reticulum in liver cells in neoplastic and preneoplastic liver cells and move into the plasma; it was termed the ‘preneoplastic antigen’ (PNA) [14]. It was later unequivocally shown that PNA is identical to mEH [15]. The presence of mEH in plasma was shown to strongly correlate with the metastasis of Kaposi’s sarcoma to the liver [16]. Recently, it has been discovered that following hepatitis C and A virus infections, mEH is also found in the plasma of patients [17]. Interestingly, these viral infections can cause the development of autoantibodies against mEH, which are believed to contribute to long-term liver damage [17]. Thus, besides research application, a sensitive method to detect mEH as a biomarker of liver damage could be useful for patients if placed by their bedside, for instance.

A variety of methods used to measure mEH levels through its enzymatic activity have been developed, including liquid chromatography with tandem mass spectrometry (LC-MS/MS) [18], radiometric assay [11], and fluorescence assay [19]. mEH protein was reported to be monitored by immunoassays [20,21,22]. Among these methods, immunoassays based on the specific binding between the antibody and antigen are the most promising for rapid and high-throughput screening and the semi-quantitative or quantitative detection of a bio-macromolecule in a patient’s biofluid. However, these antibody-based methods are limited by the low availability of the polyclonal and monoclonal antibodies, many of which show batch-to-batch variation, limited renewable supply, difficulty in epitope determination, and high production costs.

Heavy chain antibody fragments (VHHs or nanobodies), derived from camelids, are easier to produce and to standardize than monoclonal or polyclonal antibodies, thus enhancing the reproducibility and reliability of the assay. With 1/10th the size of traditional antibodies, VHHs perform comparably in affinity while having superior stability and editability, attracting rising attention as therapeutics and analytical reagents [23]. Thus, we targeted the development of an ELISA to quantify human mEH using nanobodies as the key reagent. To achieve this goal, recombinant human mEH was affinity purified and used in order to obtain human mEH-specific nanobodies from immunized llamas. Isolated VHHs were used in several formats of the sandwich-based ELISAs as well as for the detection of mEH with the Western Blot test. The usefulness and performance of the optimized assays were tested with tissue extracts from various organs.

## 2. Results and Discussion

### 2.1. The Selection of Anti-mEH Nanobodies

**Figure 1:**
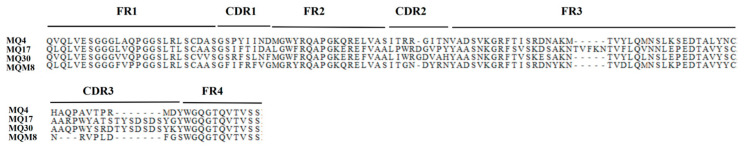
Alignments of the amino acid sequences of the four unique nanobodies against recombinant human mEH.

The expression and purification by a nickel affinity column of recombinant human mEH are described in detail in the supporting information. The purity of the target protein (>95%) was verified by SDS-PAGE (Figure S1). The recombinant human mEH was used as an immunogen. After several rounds of immunizations in llamas, a phage library of recombinant VHHs was prepared as described [24]. The diversity of the nanobody gene library was calculated to be 10^6^ based on the number of recombinant colonies after electroporation, while the library titer was 10^12^ after phage infection. To select nanobodies that recognized human mEH, three rounds of panning were conducted. Polyclonal phage ELISA was then performed to evaluate the enrichment of the positive phage clones, and the results are depicted in Figure S2. The results indicated that positive clones were effectively enriched following a second round of panning. To obtain unique nanobodies, 32 clones from the output of each round (numbers between 1–32 from the first round, 33–64 from the second round, and 65–96 from the third round) were randomly picked and cultured for the phage ELISA. As shown in Figure S3, the response of single clones differed, and the majority of them bound tightly to human mEH. After the analysis of DNA sequences, four unique positive clones called MQ4, MQ17, MQ30, and MQM8 were found. The amino acid sequence alignments are shown in Figure 1, and their frameworks were consistent with those of the llama-derived VHHs.

### 2.2. The Performance of Four Unique Nanobodies

The phagemids of the candidate nanobodies were transformed into *Escherichia coli* TOP10F’ for expression. After harvesting the bacteria, target nanobodies were extracted using B-PER Bacterial Protein Extraction and then purified with Ni-NTA resin. The purified nanobodies were confirmed using SDS-PAGE, displaying a band at approximately 17 kDa (Figure S4). The performance of each nanobody was preliminarily judged using a double antibody sandwich ELISA based on the monoclonal antibody and candidate nanobodies. As shown in Figure S5, all candidate nanobodies performed well in the double sandwich ELISA as the detection antibody. The assays were optimized and the sensitivities of the assays with the four different nanobodies are summarized in Table S1. The LODs were calculated at three times the standard deviation of the blank absorbance signal divided by the slope of the regression equation. The detection limits (LOD) of those nanobodies are within a similar range; however, the LOD sometimes exhibited a high coefficient of variation (CV) between different plates, leading to dynamic changes in the results [17]. Herein, we adjusted the slope of the regression equation to reflect the sensitivity of the assays, highlighting the situation in which the optical density increases as the concentration of the analyte increases. Although MQ30 seems to yield the worst LOD (0.91 ng mL^−1^), its sensitivity is still within the same order of magnitude as the other nanobodies tested. In addition, MQ30 showed the highest sensitivity with 0.1398 OD mL ng^−1^, which reflected that MQ30 has a higher affinity to the human mEH, and thus yields a stronger response per unit of nanobody amount. A similar result was also observed in direct ELISA by testing the titer of the candidate nanobodies (Figure S6). Taking into consideration the amount of reagent needed and experimental deviation, MQ30 was chosen for further ELISA development, although other monoclonal–nanobody and nanobody–nanobody combinations may yield better assays in the future.

Besides testing for their ability to recognize native human mEH, the selected nanobodies were assayed for their ability to recognize denatured human mEH in Western Blot assays. Recombinant human mEH was denatured and loaded onto Mini-protean TGX gels. After transferring them to a PVDF membrane, nanobodies including MQ4, MQ17, MQ30, and MQM8 were used as detection antibodies, while an HRP-labelled Anti-HA antibody was used as a reporting signal tracer. Interestingly, only VHH MQM8 was able to recognize and label the denatured mEH. It should be noted that VHH MQM8 performed the worst in the direct ELISA or double antibody sandwich ELISA, with a sensitivity of 0.0036 OD mL ng^−1^, despite having great potential to identify denatured human mEH. Hence, VHH MQM8 was chosen for further studies with the Western Blot procedure.

### 2.3. A Comparison of the Performance of Three ELISA Formats

**Scheme 1:**
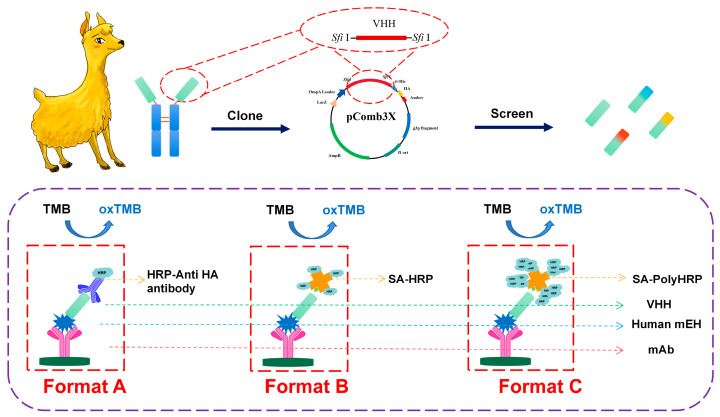
A diagram of the nanobodies as immunoprobes in ELISAs for human mEH.

**Figure 2:**
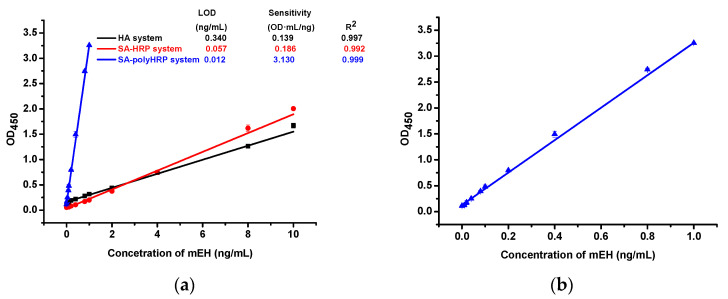
(**a**) The calibration curves of three ELISA formats by using anti-mEH monoclonal antibody (1 μg mL^−1^) as the capture antibody, native or biotinylated VHH MQ30 (1 μg mL^−1^ for format B and 0.25 μg mL^−1^ for format C) as the detection antibody, and HRP-anti-HA, SA-HRP, and SA-PolyHRP as the signal development tracer of the three kinds of formats. (**b**) The SA-PolyHRP-based format-expanded low concentration range of the analyte. Error bars indicate standard deviations (*n* = 3).

Three formats of monoclonal antibody- and nanobody-based sandwich immunoassays ware depicted in Scheme 1 by using different signal tracer strategies. Anti-human mEH monoclonal antibody was coated on the microplate as a capture antibody. Nanobodies against human mEH were used as a detection antibody in format A, while its biotinylated products were used in format B and C. HRP-anti-HA, SA-HRP, and SA-PolyHRP were applied in the three formats, respectively. The optimal concentrations of capture and detection antibodies were determined with a checkerboard assay. Standard curves of the ELISA were established and are depicted in Figure 2. Format A with an anti-HA tag antibody as a signal tracer showed a sensitivity of 0.139 OD mL ng ^−1^ and a limit of detection (LOD) of 0.340 ng mL^−1^. In format B, the assays were developed with SA-HRP as a signal tracer and reached a sensitivity of 0.186 OD mL ng^−1^ and a LOD of 0.057 ng mL^−1^. The streptavidin (SA)–biotin system could improve the assay, but the improvement was limited. Format C provided more signal responses due to its abundant HRP as a signal catalyst in a single antibody–antigen complex reaction. Due to the strong signal amplification of SA-PolyHRP, non-specific binding needed to be minimized as much as possible. Herein, the optimization of the buffer and the concentration of biotinylated VHH were investigated and depicted in Figure S7. It was found that 3% skim milk could effectively reduce non-specific binding and resulted in a lower background; it was crucial for the format C system due to the powerful amplification capability of PolyHRP. Under an optimal condition, format C offered a sensitivity of 3.130 OD mL ng^−1^ and a LOD of 0.012 ng mL^−1^. The introduction of format B showed a 5.9-fold lower LOD and a 1.3-fold higher sensitivity than format A. Format C with SA-polyHRP performed excellently with a nearly 28-fold higher sensitivity and 22-fold lower LOD than format A.

### 2.4. Cross-Reactivity

The selectivity of the proposed methods was evaluated by the ELISA spiked with mEH from rat mEH and other human epoxide hydrolases. PBS was spiked with 1000 ng mL^−1^ of different epoxide hydrolases, and the detected concentrations of the spiked samples were calculated according to the standard plot of the human mEH. The cross-reactivities are reported in Table 1. The assay showed slight cross-reactivity with denatured mEH (0.02%) and rat mEH (0.11%). The latter result is quite surprising, as the human and rat mEH were reported to be over and around 80% similar [25]. Marginal cross-reactivity was observed for human sEH, human EH-3, and human EH-4. Given the low identity reported between human mEH and the other human epoxide hydrolases [26], these results are not surprising. The methods proposed demonstrated that the Mab-VHH ELISA was highly specific to the human mEH.

### 2.5. Matrix Effects

To evaluate the practicality of the assays, the performances of the calibration curve in clinical samples were studied. Sample dilution is the most common way to eliminate the matrix effect, whereas there are more requirements for the sensitivity of the assays. Herein, spike-recovery tests were applied to verify the matrix effects of the assays in clinical samples. Three kinds of experiments were conducted with a 1:10, 1:100, and 1:1000 dilution of the human plasma and liver tissue sample from mice spiked with human mEH (see Table 2 and Table S2). The recoveries were 77–120%, 74–121%, and 73–126% for 1:10, 1:100, and 1:1000, respectively. It was noted that although the CR of the murine mEH was low, the mEH was detectable in assays because mouse liver has a high mEH resulting in high levels of mEH even with the 1:100 dilution of the mouse liver homogenates. In terms of tissue extraction, taking format C as an example, the recovery was 72–141%, 72–88%, and 79–108% for 1:10, 1:100, and 1:1000, respectively. It was noted that the 1:10 dilution showed a certain matrix effect with relatively poor recoveries comparing with higher dilutions, but it was still acceptable for the detection of human mEH. Overall, these data support the reliability of human mEH ELISAs in practical applications, indicating the assays based on Mab-VHH ELISAs were promising and showed potential in the analysis of practical samples. Table 2 shows the accuracy and precision analysis of human mEH in spiked human plasma samples by different format immunoassays.

### 2.6. The Analysis of Human Tissue Samples

Mab-VHH ELISAs were applied to detect human mEH in human tissue samples. Three kinds of ELISAs, the proposed Western Blot, and enzyme activity were used to analyze the same samples. All samples were diluted 10^3^, 10^4^, and 10^5^ times, and they were applied in ELISAs. The original sample extractions were loaded onto the gel for the Western Blot by using 1 μg mL^−1^ MQM8-HRP as a signal tracer. As shown in Table 3 and Figure S8, the results of the ELISAs were comparable to the enzyme activity based on the radiometric assay by using ^3^H *c*-SO as substrate (correlation *R*^2^ > 0.95). The results of the Western Blot by using VHH MQM8 as a detection antibody are displayed in Figure S9. While a clear single band can be observed in pure recombinant mEH, multiple bands are noticeable in crude extract samples when using MQM8 as the primary antibody in Western blotting. This indicates nonspecific binding. Despite observing a distinct stronger band at the right molecular weight in liver samples, whether it corresponded to the mEH protein band remained uncertain. In the other extracts, we speculated the band near the recombinant protein band might be the mEH, but we could not be certain. Therefore, MQM8 detection capability in the Western Blot is limited to recombinant protein and not crude extracts. On the other end, the ELISAs were effective at recognizing the bioactive mEH in the crude extracts, underlying the usefulness of this analytical format using nanobodies.

## 3. Materials and Methods

### 3.1. Materials

The anti-human mEH monoclonal antibody (1H9) was prepared as described in the previous work [17]. Electrocompetent *E. coli* ER2738 cells were purchased from the Lucigen Corporation (Middleton, WI, USA). HRP tag Anti-M13 Major Coat Protein Antibody (RL-ph1) (RRID: AB_673750) was purchased from Santa Cruz Biotechnology, Inc. (Dallas, TX, USA). The anti-HA tag antibody with HRP was purchased from Roche (RRID: AB_390917) (Branchburg, NJ, USA). Streptavidin PolyHRP40 conjugate (SA-PolyHRP) was purchased from Fitzgerald Industries International (Concord, MA, USA). LeukoLOCK Total RNA Isolation System, TOP10F’ Chemically Competent cells of *E. coli*, B-PER™ bacterial protein extraction reagent, and HisPur™ Ni-NTA Resin were purchased from Thermo Fisher Scientific (Rockford, IL, USA). Streptavidin-HRP conjugate (SA-HRP) was purchased from Southern Biotech (Birmingham, AL, USA). Plasma from human blood, sodium periodate, sulfo-NHS-LC-biotin and 3,3′,5,5′-tetramethylbenzidine (TMB) were obtained from Sigma-Aldrich (St. Louis, MO, USA). DNA sequencing was conducted by the UC Davis DNA Sequencing Facility. Unless otherwise specified, all other chemicals and reagents used were of analytical grade. 

### 3.2. Expression and Purification of Human mEH in Tni Insect Cells

The cDNA coding for human microsomal epoxide hydrolase (mEH) (Uniprot: P07099) was subcloned into a baculovirus transfer vector, pAcUW21, with slight modifications. For purification and subsequent affinity tag removal, the C-terminus of mEH was modified to include a Factor Xa protease site, short spacer, and His6 affinity tag. Specifically, the final four amino acid residues of mEH (LERQ) were mutated to the Factor Xa sequence (IEGR), followed by a GGSG spacer, and six histidine residues. The modified mEH gene sequence was PCR amplified, subcloned into pAcUW21, and verified with Sanger Sequencing (UC Davis DNA Sequencing Facility). The verified mEH pAcUW21 vector was then transfected into Sf9 (Spodoptera frugiperda) insect cells using a baculovirus transfection kit (Expression Systems, LLC). Baculoviruses were generated and viral supernatant was harvested after 5 days. The viral supernatant was subsequently used to transduce a larger volume of Sf9 insect cells to amplify the virus. This procedure was repeated once more to generate a high titer baculovirus termed P2. For human mEH protein expression, 500 mL cultures of Tni (Trichoplusia ni) insect cells at a density of 1.5 × 10^6^ cells/mL were transduced with approximately 1 mL of P2 viral supernatant. Cells were grown at 27 °C, 125 rpm, for 72 h. At 72 h, cell viability was checked using an automated cell counter. Cells were harvested by centrifugation when cell viability was approximately 75%. 

For purification, cell pellets were resuspended in 10 volumes (10 mL/1 g cells) of homogenization buffer (50 mM Tris, pH 8.0, 300 mM NaCl, 250 mM sucrose, 10% glycerol). Cells were homogenized by stirring at 4 °C for at least one hour or until homogeneous. The cells were lysed on ice using a mechanical cell homogenizer (4 cycles of 30 s each, with one minute of rest between each cycle). Cellular debris was cleared by centrifugation at 16,500 rpm, 4 °C, 30 min. Next, cellular microsomes were isolated from the lysate by centrifugation at 100,000× *g*, 4 °C, two hours. After centrifugation, cell lysate was carefully decanted without disturbing the pelleted microsomal fraction. All cellular microsomes were resuspended together in extraction buffer (50 mM Tris, pH 8.5, 150 mM NaCl, 10 mM imidazole, 10% glycerol, 0.5% β-DDM). Microsomes were solubilized with stirring at 4 °C, three hours. Insoluble debris was removed by another round of centrifugation at 100,000× *g*, 4 °C, 30 min. 

Solubilized cell microsomes were than incubated with approximately 2 mL Ni-NTA beads which had been equilibrated in equilibration buffer (50 mM Tris, pH 8.5, 150 mM NaCl, 10 mM imidazole, 0.05% β-DDM) at 4 °C for at least one hour. After incubation, the Ni-NTA beads and microsomal fraction were loaded into a gravity column and washed with buffer containing 50 mM Tris, pH 8.5, 150 mM NaCl, 60 mM imidazole, 0.05% β-DDM. Next, protein was eluted from the column using the same buffer containing 300 mM imidazole. Fractions corresponding to one column volume were collected and analyzed using UV-Vis spectroscopy until the A280 measurement was close to 0. Fractions were collected, concentrated, then further purified by size exclusion chromatography. Briefly, 1 mL of purified protein was injected into a Bio-Rad NGC FPLC system equipped with a Bio-Rad Enrich High-Resolution Size Exclusion column. Protein was eluted in a buffer containing 50 mM Tris, pH 8.5, 150 mM NaCl, 5% glycerol, 0.05% β-DDM. Peak fractions were analyzed by SDS-PAGE and pure fractions were concentrated to a volume of approximately 0.5 mL. Protein concentration was tested using a standard BCA assay and aliquots were stored at −80 °C until use.

### 3.3. The Construction of a Nanobody Library and Bio-Panning

A blood sample was obtained from a 3-year-old llama that had undergone immunization with six injections of recombinant human microsomal epoxide hydrolase (mEH) at a dose of 500 μg per injection, with an interval of 14 days between each injection, administered with incomplete Freund’s adjuvant. The total RNA was isolated from llama blood after the fifth immunization, extracted by LeukoLOCK total RNA isolation system and stored at −80 °C until used. First-strand complementary DNA (cDNA) was generated from total RNA by using the SuperScript first-strand synthesis system according to the manufacturer’s instructions. Phage library construction was prepared based on the detailed protocol described by Carlos F. Barbas [27]. Three rounds of panning were conducted by separately coating 20, 10, and 5 μg mL^−1^ recombinant human mEH on the microplate. After blocking with 3% skim milk, a 100 μL/well VHH phage library was added into the microplate at RT for 1 h. Unbound and weakly bound phage particles were removed by PBST washing. Then, a 100 μL/well 0.1 M glycine-HCl (pH = 2.0) was added and incubated for 10 min to elute the binding phages; a 50 μL/well 1 M Tris-HCl (pH = 8.8) was subsequently added to neutralize the elution buffer. Polyclonal phage ELISA was analyzed to evaluate the effectiveness of the panning overall. Single clones were randomly chosen and tested by phage ELISA. Positive clones were cultured and sequenced for further study.

### 3.4. The Expression and Purification of Nanobodies

The positive phagemids were extracted and then transferred into *E. coli* TOP 10F’ cells using heat shock. A single colony was picked and cultured in 10 mL of a Super Broth (SB) medium with 50 µg mL^−1^ carbenicillin. An amount of 1 mL of the overnight culture was added to 100 mL of SB medium containing 50 µg mL^−1^ carbenicillin. When the culture reached an OD_600_ value of 0.8, IPTG was added to a final concentration of 0.05 M, and the culture was shaken at 30 °C overnight. The bacterial pellets were lysed with a B-PER lysis buffer for 1 h, and the lysate supernatants were collected through centrifugation at 13,000× *g* for 10 min, followed by purification through a Ni-NTA resin column. The column was equilibrated and washed with 10 mM imidazole after loading it with the supernatants. The captured nanobodies were then eluted with 250 mM imidazole. The purified VHHs were dialyzed against PBS and verified by SDS-PAGE.

### 3.5. The Development of Three Formats of Nanobody-Based Immunoassays for the Detection of Human mEH

Three kinds of immunoassay formats based on the mAb and nanobody sandwich were analyzed and applied in the detection of human mEH. A biotinylation of human mEH nanobodies (MQ4, MQ17, MQ30, MQM8) was conducted according to the description of Dongyang Li et al. [28]. ELISAs were conducted as previously described with minor modifications. Briefly, a 100 µL/well (1 µg mL^−1^) mAb was coated on the microplate at 4 °C overnight. After blocking with 3% skim milk, a series of human mEH dilutions (100 µL/well) were added and incubated at RT for 1 h. After washing with PBST, nanobodies with or without a biotin tag were added and incubated at RT for 1 h. Then, different signal tracers were added in appropriate formats and incubated at RT for another 1 h. After the final washing, color development using TMB substrate (100 μL/well) was allowed to proceed for 15 min. The reaction was stopped by 2 M H_2_SO_4_ (50 μL/well), and the absorbance was measured at 450 nm in a microplate reader (SpectraMax^®^ M2, Molecular Devices, San Jose, CA, USA).

### 3.6. Cross-Reactivity

The selectivity of the proposed ELISA was applied to detect a group of epoxide hydrolases. Different epoxide hydrolases including the mEHs from other species and other human epoxide hydrolases expressed in a previous work [11] were spiked at a final concentration of 1000 ng mL^−1^ in PBS. The prepared enzymes were then tested in a format A immunoassay along with human mEH standard solutions. Cross reactivities (CR) were calculated by the equation as follows: CR (%) = [the measured concentration of the analogs/spiked concentration of the analogs] × 100%.

### 3.7. Matrix Effects

Different formats of ELISAs were utilized for liver tissue samples from mice and human plasma. A series of dilutions was set to evaluate the matrix effects of the methods. In brief, liver tissue extraction or human plasma samples with different dilutions (1:10, 1:100, 1:1000) in 0.1% BSA in PBS buffer were spiked with a series standard of human mEH and then tested by the ELISAs. An anti-mEH monoclonal antibody (1 μg mL^−1^) was used as the capture antibody; native or biotinylated VHH MQ30 (1 μg mL^−1^) was used as the detection antibody; and HRP-anti-HA, SA-HRP, and SA-PolyHRP was used as the signal development tracer of the immunoassays. The recoveries of the human mEH were calculated to verify the matrix effect of the proposed methods.

### 3.8. Analysis of Clinical Samples

Seven kinds of whole tissue extract were obtained from human commercial tissue samples (BioChain Institute, Newark, CA, USA). The samples were tested by all three formats of the proposed immunoassays at a dilution of 10^3^, 10^4^, and 10^5^-fold and verified by monitoring the enzyme activity test and with a Western Blot using nanobody MQM8.

## 4. Conclusions

In summary, anti-human mEH-specific nanobodies were obtained, expressed, and purified. These nanobodies performed excellently in the development of the double antibody sandwich ELISA paired with a monoclonal antibody. By introducing SA-PolyHRP, the assay provided a sensitivity of 3.130 OD mL ng^−1^ and LOD of 0.012 ng mL^−1^ in human mEH detection, with 28-fold higher sensitivity and 22-fold lower LOD than the ‘conventional’ one (using HRP-labeled Anti-HA tag antibody as a signal tracer) without amplification. Surprisingly, one of the candidate nanobodies named MQM8 showed a recognition ability for denatured human mEH, which resulted in the feasible development of a Western Blot. The matrix effect was studied in human plasma and in mice liver samples using a spike-recovery test. All formats with different signal trigger strategies performed well with clinical tissue samples. Both ELISA and Western Blot were comparable with the enzyme activity tests of the human tissue samples. Although MQM8 demonstrated excellent performance in detecting denatured recombinant mEH in a Western Blot format, it detected numerous protein bands when applied to the analysis of organs’ crude extracts. This makes it unsuitable as a reliable Western Blot detection reagent. Nevertheless, nanobodies act as promising immune probes in ELISA human mEH and can be suitable for the monitoring of the clinical diagnosis and prognosis of disease. Regarding diagnosis usage, mEH was found many years ago to dissociate from the endoplasmic reticulum in liver cells in neoplastic and preneoplastic cells and move into the plasma. In this context, the mEH protein was termed the ‘preneoplastic antigen’ (PNA) [14,15]. By labeling a selective substrate of the mEH with carrier free tritium, a very sensitive assay was developed for mEH in plasma [16]. This assay demonstrated that the presence of mEH in plasma was highly correlated with the metastasis of Kaposi’s sarcoma in the liver. Although an apparently good biomarker of liver damage and complementary to other hepatic markers such as alpha fetoprotein, mEH was not fully evaluated as a biomarker due to a reluctance to use radioactive assays clinically. Although an earlier ELISA for mEH was developed, it was not sensitive enough for clinical use [22]. Possibly a highly sensitive, ‘immortal nanobody’-based assay will warrant a reexamination of mEH or preneoplastic antigen in diagnoses.

## Figures and Tables

**Table 1:** The specificity of the nanobody (MQ30)-based ELISA for human mEH with mEH from other species and other human epoxide hydrolases. Error bars indicate standard deviations (*n* = 3).

Epoxide Hydrolases	Spiked Concentration (ng mL^−1^)	Measured Concentration Mean ± SD (ng mL^−1^)	Cross-Reactivity (%)
Denatured Meh ^a^	1000	0.21 ± 0.06	0.02
Rat mEH	1000	1.05 ± 0.28	0.11
Human sEH	1000	N.D.	<0.01
Human EH-3	1000	N.D.	<0.01
Human EH-4	1000	N.D.	<0.01

^a^ Denatured mEH was treated with 2% SDS and then heated to 95 °C for 10 min.

**Table 2:** Accuracy and precision analysis of human mEH in spiked human plasma samples by different format immunoassays.

Format A												
	**Intra-Assay**						**Inter-Assay**					
**Spiked** **(ng mL^−1^)**	**1:10 Dilution**		**1:100 Dilution**		**1:1000 Dilution**		**1:10 Dilution**		**1:100 Dilution**		**1:1000 Dilution**	
	**Found** **(ng mL^−1^)**	**Recovery** **(%)**	**Found** **(ng mL^−1^)**	**Recovery** **(%)**	**Found** **(ng mL^−1^)**	**Recovery** **(%)**	**Found** **(ng mL^−1^)**	**Recovery** **(%)**	**Found** **(ng mL^−1^)**	**Recovery** **(%)**	**Found** **(ng mL^−1^)**	**Recovery** **(%)**
8.00	6.48 ± 0.81	81	8.21 ± 0.25	103	7.65 ± 0.38	96	6.14 ± 0.17	77	7.86 ± 0.82	98	6.53 ± 0.63	82
4.00	4.53 ± 0.31	113	4.59 ± 0.23	115	4.53 ± 0.08	113	4.65 ± 0.12	116	4.66 ± 0.15	117	4.50 ± 0.12	112
2.00	2.02 ± 0.11	101	2.38 ± 0.06	119	2.35 ± 0.04	118	2.08 ± 0.08	104	2.20 ± 0.13	110	2.12 ± 0.18	106
1.00	0.86 ± 0.07	86	0.96 ± 0.09	96	1.28 ± 0.10	105	0.87 ± 0.06	87	1.15 ± 0.01	115	1.20 ± 0.07	120
0.50	0.57 ± 0.01	115	0.59 ± 0.13	118	0.46 ± 0.01	93	0.65 ± 0.04	130	0.58 ± 0.09	115	0.55 ± 0.16	110
Format B												
	**Intra-Assay**						**Inter-Assay**					
**Spiked** **(ng mL** **^−1^)**	**1:10 Dilution**		**1:100 Dilution**		**1:1000 Dilution**		**1:10 Dilution**		**1:100 Dilution**		**1:1000 Dilution**	
	**Found** **(ng mL** **^−1^)**	**Recovery** (%**)**	**Found** **(ng mL** **^−1^)**	**Recovery** **(%)**	**Found** **(ng mL** **^−1^)**	**Recovery** **(%)**	**Found** **(ng mL** **^−1^)**	**Recovery** **(%)**	**Found** **(ng mL** **^−1^)**	**Recovery** **(%)**	**Found** **(ng mL** **^−1^)**	**Recovery** **(%)**
8.00	6.90 ± 0.46	86	8.23 ± 0.30	103	8.30 ± 0.61	104	6.07 ± 0.23	76	7.17 ± 0.17	90	7.18 ± 0.28	90
4.00	4.95 ± 0.01	124	4.81 ± 0.03	120	4.81 ± 0.19	120	4.79 ± 0.32	120	4.73 ± 0.14	118	4.83 ± 0.13	121
2.00	2.30 ± 0.13	115	2.23 ± 0.09	112	2.37 ± 0.10	118	1.71 ± 0.09	85	2.29 ± 0.10	114	2.35 ± 0.22	117
1.00	0.99 ± 0.05	99	1.05 ± 0.10	105	1.22 ± 0.04	122	0.74 ± 0.05	74	0.81 ± 0.03	81	1.20 ± 0.15	120
0.50	0.45 ± 0.13	90	0.42 ± 0.07	83	0.49 ± 0.05	98	0.37 ± 0.04	75	0.41 ± 0.08	82	0.40 ± 0.06	80
Format C												
	**Intra-Assay**						**Inter-Assay**					
**Spiked** **(ng mL** **^−1^)**	**1:10 Dilution**		**1:100 Dilution**		**1:1000 Dilution**		**1:10 Dilution**		**1:100 Dilution**		**1:1000 Dilution**	
	**Found** **(ng mL** **^−1^)**	**Recovery** **(%)**	**Found** **(ng mL** **^−1^)**	**Recovery** **(%)**	**Found** **(ng mL** **^−1^)**	**Recovery** **(%)**	**Found** **(ng mL** **^−1^)**	**Recovery** **(%)**	**Found** **(ng mL** **^−1^)**	**Recovery** **(%)**	**Found** **(ng mL** **^−1^)**	**Recovery** **(%)**
1.00	0.75 ± 0.02	75	0.84 ± 0.06	84	0.75 ± 0.02	75	0.72 ± 0.02	72	0.76 ± 0.03	76	0.77 ± 0.05	77
0.80	0.60 ± 0.03	75	0.75 ± 0.02	94	0.63 ± 0.01	79	0.58 ± 0.01	73	0.60 ± 0.01	75	0.59 ± 0.05	73
0.40	0.33 ± 0.02	83	0.39 ± 0.03	98	0.35 ± 0.02	88	0.28 ± 0.01	70	0.39 ± 0.02	98	0.39 ± 0.03	96
0.20	0.19 ± 0.01	93	0.20 ± 0.01	101	0.18 ± 0.03	90	0.20 ± 0.02	98	0.20 ± 0.02	118	0.21 ± 0.02	103
0.10	0.08 ± 0.01	83	0.09 ± 0.01	86	0.10 ± 0.02	104	0.11 ± 0.01	113	0.13 ± 0.01	126	0.10 ± 0.01	99

**Table 3:** A comparison of nanobody-based ELISA and other methods for the analysis of human mEH in the human tissue whole cell extract samples.

Human Tissues	mEH Concentration (nM) ^a^			
	Enzyme Activity ^b^	HRP-Anti-HA Antibody ELISA	SA-HRP ELISA	SA-Poly HRP ELISA
Adrenal	290 ± 50	159 ± 11	172 ± 10	136 ± 8
Esophagus	12 ± 1	N.D. ^c^	3 ± 0.4	2 ± 0.03
Liver	850 ± 50	292 ± 9	322 ± 8	261 ± 24
Pancreas	67 ± 4	17 ± 0.6	20 ± 1.0	21 ± 2
Ovary	6 ± 1	N.D.	N.D.	0.1 ± 0.05
Heart	10 ± 4	N.D.	N.D.	0.2 ± 0.1
Hippocampus	17 ± 2	8 ± 0.2	7 ± 0.1	6 ± 0.7

^a^ Results are average ± SD (*n* = 3); 1 nM mEH corresponds to 50 ng mL^−1^ mEH; All samples were diluted in 10^3^, 10^4^ and 10^5^ for ELISA. ^b^ Data from ref. [11]. ^c^ N.D. not detection.

## Data Availability

Data in this study are available from the corresponding authors upon request.

## Supplementary Materials

The supporting information can be downloaded at: https://www.mdpi.com/article/10.3390/ijms241914698/s1.

## References

[1] Vaclavikova R., Hughes D.J., Soucek P. (2015). Microsomal epoxide hydrolase 1 (EPHX1): Gene, structure, function, and role in human disease. Gene.

[2] Omiecinski C.J., Aicher L., Swenson L. (1994). Developmental expression of human microsomal epoxide hydrolase. J. Pharmacol. Exp. Ther..

[3] Decker M., Arand M., Cronin A. (2009). Mammalian epoxide hydrolases in xenobiotic metabolism and signalling. Arch. Toxicol..

[4] Fritz P., Mürdter T.E., Eichelbaum M., Siegle I., Weissert M., Zanger U.M. (2001). Microsomal epoxide hydrolase expression as a predictor of tamoxifen response in primary breast cancer: A retrospective exploratory study with long-term follow-up. J. Clin. Oncol..

[5] Lee W.J., Brennan P., Boffetta P., London S.J., Benhamou S., Rannug A., To-Figueras J., Ingelman-Sundberg M., Shields P., Gaspari L. (2002). Microsomal epoxide hydrolase polymorphisms and lung cancer risk: A quantitative review. Biomarkers.

[6] Zusterzeel P.L., Peters W.H., Visser W., Hermsen K.J., Roelofs H.M., Steegers E.A. (2001). A polymorphism in the gene for microsomal epoxide hydrolase is associated with pre-eclampsia. J. Med. Genet..

[7] Liu M., Sun A., Shin E.J., Liu X., Kim S.G., Runyons C.R., Markesbery W., Kim H.C., Bing G. (2006). Expression of microsomal epoxide hydrolase is elevated in Alzheimer’s hippocampus and induced by exogenous beta-amyloid and trimethyl-tin. Eur. J. Neurosci..

[8] Gautheron J., Morisseau C., Chung W.K., Zammouri J., Auclair M., Baujat G., Capel E., Moulin C., Wang Y., Yang J. (2021). EPHX1 mutations cause a lipoatrophic diabetes syndrome due to impaired epoxide hydrolysis and increased cellular senescence. eLife.

[9] Edin M.L., Hamedani B.G., Gruzdev A., Graves J.P., Lih F.B., Arbes S.J., Singh R., Orjuela Leon A.C., Bradbury J.A., DeGraff L.M. (2018). Epoxide hydrolase 1 (EPHX1) hydrolyzes epoxyeicosanoids and impairs cardiac recovery after ischemia. J. Biol. Chem..

[10] Calder P.C. (2004). n–3 Fatty acids and cardiovascular disease: Evidence explained and mechanisms explored. Clin. Sci..

[11] Morisseau C., Kodani S.D., Kamita S.G., Yang J., Lee K.S.S., Hammock B.D. (2021). Relative Importance of Soluble and Microsomal Epoxide Hydrolases for the Hydrolysis of Epoxy-Fatty Acids in Human Tissues. Int. J. Mol. Sci..

[12] Edin M.L., Zeldin D.C. (2021). Regulation of cardiovascular biology by microsomal epoxide hydrolase. Toxicol. Res..

[13] Marowsky A., Meyer I., Erismann-Ebner K., Pellegrini G., Mule N., Arand M. (2017). Beyond detoxification: A role for mouse mEH in the hepatic metabolism of endogenous lipids. Arch. Toxicol..

[14] Lin J.-C., Hiasa Y., Farber E. (1977). Preneoplastic Antigen as a Marker for Endoplasmic Reticulum of Putative Premalignant Hepatocytes during Liver Carcinogenesis. Cancer Res..

[15] Griffin M.J., Noda K. (1980). Quantitation of Epoxide Hydrolase Released from Hyperplastic Nodule and Hepatoma Microsomes. Cancer Res..

[16] Hammock B.D., Loury D.N., Moody D.E., Ruebner B., Baselt R., Milam K.M., Volberding P., Ketterman A., Talcott R. (1984). A methodology for the analysis of the preneoplastic antigen. Carcinogenesis.

[17] Akatsuka T., Kobayashi N., Ishikawa T., Saito T., Shindo M., Yamauchi M., Kurokohchi K., Miyazawa H., Duan H., Matsunaga T. (2007). Autoantibody response to microsomal epoxide hydrolase in hepatitis C and A. J. Autoimmun..

[18] Wu T., Xi X., Chen Y., Jiang C., Zhang Q., Dai G., Bai Y., Zhang W., Ni T., Zou J. (2021). Absolute protein assay for the simultaneous quantification of two epoxide hydrolases in rats by mass spectrometry-based targeted proteomics. J. Sep. Sci..

[19] Morisseau C., Bernay M., Escaich A., Sanborn J.R., Lango J., Hammock B.D. (2011). Development of fluorescent substrates for microsomal epoxide hydrolase and application to inhibition studies. Anal. Biochem..

[20] Duan H., Yoshimura K., Kobayashi N., Sugiyama K., Sawada J., Saito Y., Morisseau C., Hammock B.D., Akatsuka T. (2012). Development of monoclonal antibodies to human microsomal epoxide hydrolase and analysis of “preneoplastic antigen”-like molecules. Toxicol. Appl. Pharmacol..

[21] Backman J.T., Siegle I., Zanger U.M., Fritz P. (1999). Immunohistochemical detection of microsomal epoxide hydrolase in human synovial tissue. Histochem. J..

[22] Gill S.S., Wie S.I., Guenthner T.M., Oesch F., Hammock B.D. (1982). Rapid and sensitive enzyme-linked immunosorbent assay for the microsomal epoxide hydrolase. Carcinogenesis.

[23] Liu M., Li L., Jin D., Liu Y. (2020). Nanobody-A versatile tool for cancer diagnosis and therapeutics. Wiley Interdiscip. Rev. Nanomed. Nanobiotechnol..

[24] Cui Y., Li D., Morisseau C., Dong J.X., Yang J., Wan D., Rossotti M.A., Gee S.J., González-Sapienza G.G., Hammock B.D. (2015). Heavy chain single-domain antibodies to detect native human soluble epoxide hydrolase. Anal. Bioanal. Chem..

[25] Skoda R.C., Demierre A., McBride O.W., Gonzalez F.J., Meyer U.A. (1988). Human microsomal xenobiotic epoxide hydrolase. Complementary DNA sequence, complementary DNA-directed expression in COS-1 cells, and chromosomal localization. J. Biol. Chem..

[26] Decker M., Adamska M., Cronin A., Di Giallonardo F., Burgener J., Marowsky A., Falck J.R., Morisseau C., Hammock B.D., Gruzdev A. (2012). EH3 (ABHD9): The first member of a new epoxide hydrolase family with high activity for fatty acid epoxides. J. Lipid Res..

[27] Barbas C.F., Burton D.R., Scott J.K., Silverman G.J. (2001). Phage Display: A Laboratory Manual.

[28] Li D., Cui Y., Morisseau C., Gee S.J., Bever C.S., Liu X., Wu J., Hammock B.D., Ying Y. (2017). Nanobody Based Immunoassay for Human Soluble Epoxide Hydrolase Detection Using Polymeric Horseradish Peroxidase (PolyHRP) for Signal Enhancement: The Rediscovery of PolyHRP?. Anal. Chem..

