# Supplementary material for: The Generation of a Nanobody-Based ELISA for Human Microsomal Epoxide Hydrolase
Source: Int J Mol Sci. 2023 Sep 28;24(19):14698. doi: 10.3390/ijms241914698 (PMC10572367; doi:10.3390/ijms241914698)
Supplement: Supplementary file 1 [file ijms-24-14698-s001.zip › ijms-2585417-supplementary.pdf]

Electronic Supplementary Material

The generation of a Nanobody-based ELISA for the Human Microsomal Epoxide Hydrolase

Qiyi He [a,b], Mark R. McCoy [a], Meng Qi [a], Christophe Morisseau [a], Huiyi Yang [a,b], Chengpeng Xu [a], Rachel Shey [a], Michael C. Goodman [a], Suqing Zhao [b,*], Bruce D. Hammock [a,*]

[a] *Department of Entomology and Nematology, and UCD Comprehensive Cancer Center, University of California, Davis, Davis, CA 95616, USA*

[b] *Department of Pharmaceutical Engineering, School of Biomedical and Pharmaceutical Sciences, Guangdong University of Technology, Guangzhou 510006, China*

* Correspondence: sqzhao@gdut.edu.cn (S.Z.); bdhammock@ucdavis.edu (B.D.H.)

Western blot for human tissues samples by using VHH MQM8

Briefly, recombinant human mEH or clinical samples (~3mg mL^{-1} of each human tissues extractions) were mixed with loading buffer, and heated at 95°C for 10min. 10 μL of the mixtures were loaded into the mini-protein TGX precast gel (4-15%) and run for 32 min at 200 V. The gel was then assembled to the filter along with the PVDF membrane for the transformation through Bio-Rad trans-blot turbo transfer system under the instructions by the manufacturer's protocol. After 10 min trans blotting, the membrane was picked and blocked with EveryBlot blocking buffer (Cat:12010020) for 5 min. Next, 1 μg mL^{-1} MQM8-HRP in EveryBlot blocking buffer was added and incubated for 1h. After five times washing, ECL substrates solutions were mixed and spread onto the membrane and images were captured by the software ImageLab 5.0. Software.

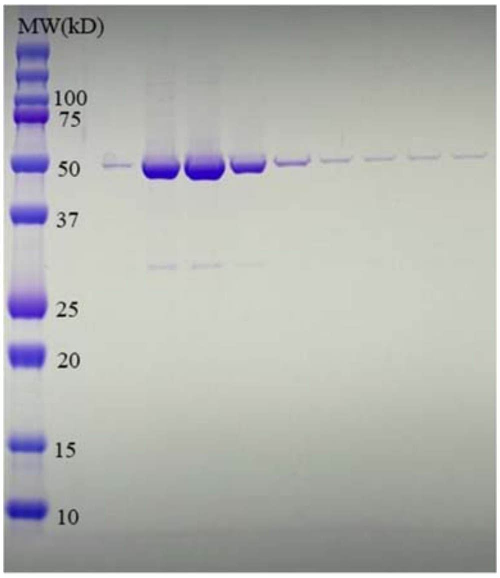

Figure S1. SDS-PAGE result for the recombinant human mEH with Factor Xa. SDS-PAGE analysis of fractions collected from the Nickel affinity purification. The factor Xa was not digested yet.

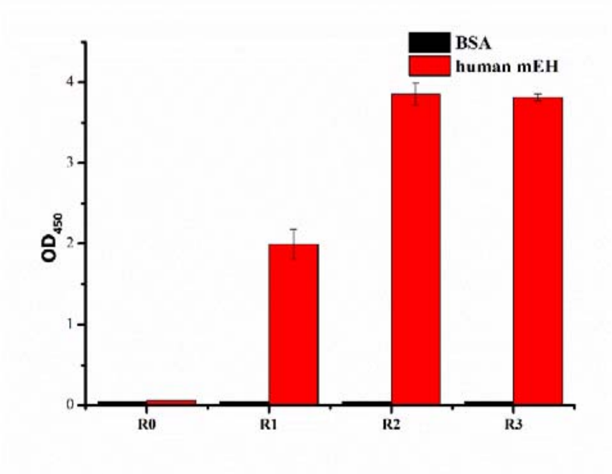

Figure S2. Polyclonal phage ELISA analysis of the enrichment of specific phage-displayed VHH after three rounds panning.

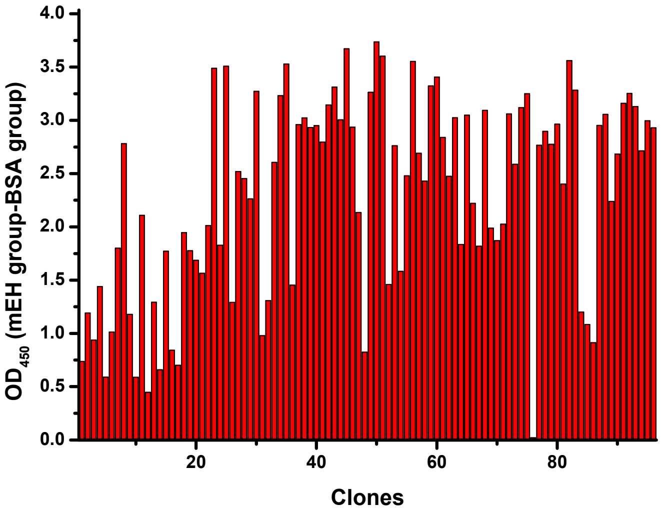

Figure S3. Evaluation of 96 randomly selected individual phage-displayed VHHs in ELISA.

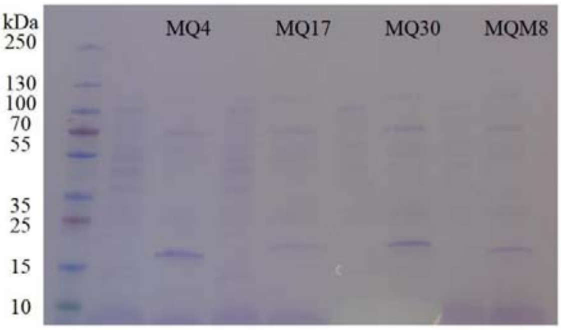

Figure S4. SDS-PAGE analysis of affinity purified nanobodies.

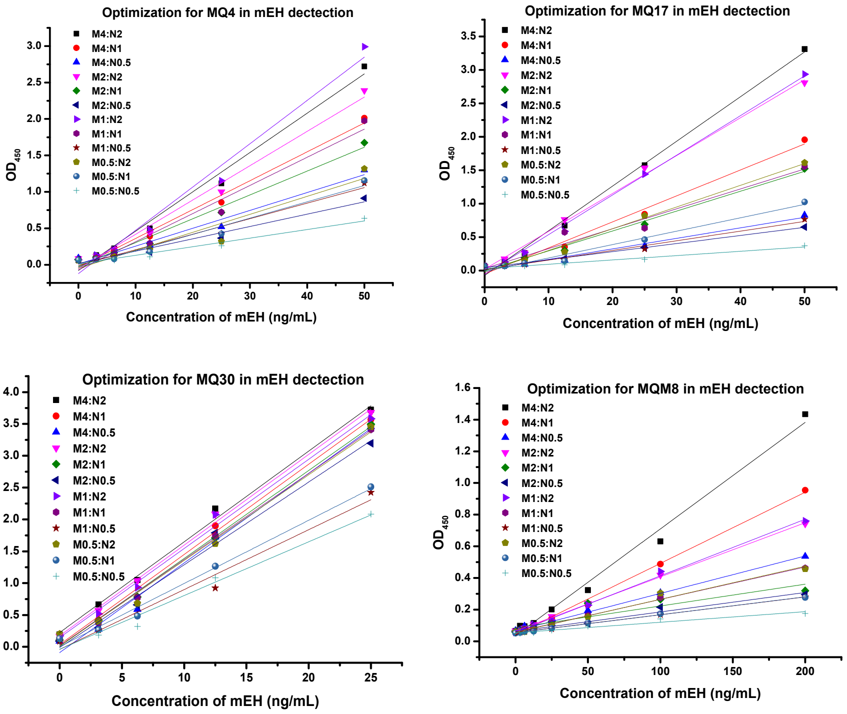

Figure S5. The optimization of monoclonal antibody and different nanobodies based sandwich ELISA for human mEH. The assays were conducted by using different concentrations of anti-mEH mAb (eg. M1 stands for mAb at 1 μg mL^{-1}) as the capture antibody, native VHH (eg. N1 stands for nanobody at 1 μg mL^{-1}) as the detection antibody, and HRP-anti-HA as the signal development.

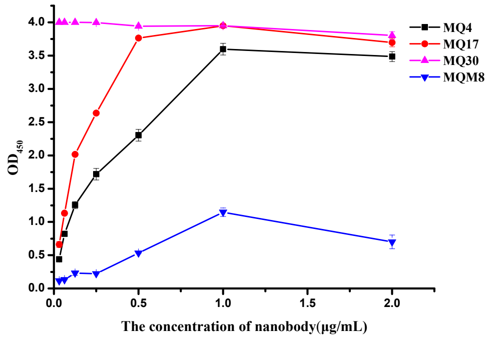

Figure S6. The result of direct ELISA by coating 0.5 μg/mL recombinant human mEH on the microplate and using a series concentration of candidate nanobody as detection antibody. HRP labeled Anti-HA tag antibody was then added as secondary antibody for signal development. Error bars indicate standard deviations (*n* = 3).

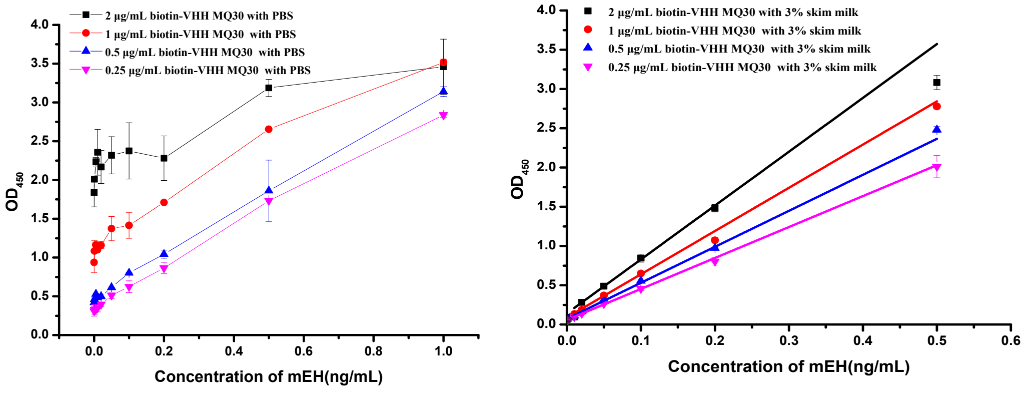

(a) **(b)**

Figure S7. Calibration curves of SA-polyHRP based ELISAs (format C) with varying concentrations of the detection antibody (biotin-VHH MQ30, 0.25-2.00 µg mL^{-1}) in (a) PBS or (b) 3% SM/PBS. Error bars indicate standard deviations (n = 3).

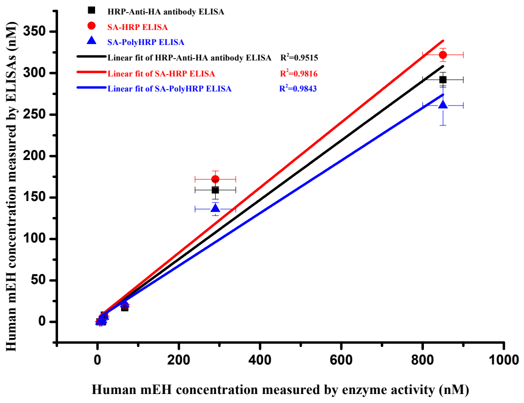

Figure S8. Correlation of the ELISAs and the enzyme activity based on the radiometric method using *c*-SO as substrate. The data are plotted from Table 2 and generated from whole tissue extracts derived from 7 different tissues.

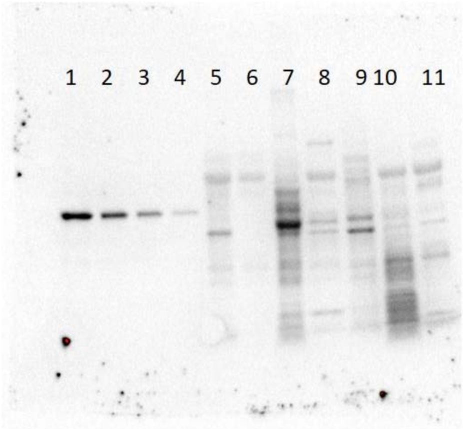

Figure S9. Western blot results by using MQM8 nanobody for human mEH. Lane 1-4: 1, 0.5, 0.2, 0.1 μg human recombinant mEH (from left to right); Lane 5: adrenal; Lane 6: ovary; Lane 7: liver; Lane 8, heart; Lane 9, hippocampus; Lane 10: pancreas; Lane 11: esophagus.

Table S1. Summary of the performance of the different strains of nanobody based immunoassay for human mEH under the optimal condition.

Types	Sensitivity (OD mL ng)	LOD (ng mL^{-1})
MQ4	0.0619	0.51
MQ17	0.0608	0.18
MQ30	0.1398	0.91
MQM8	0.0036	0.30

Table S2 Recovery test of human mEH spiked into the liver tissues sample from mice (*n* = 3) by different format immunoassays.

Format A						
Spiked ($ng\ mL^{-1}$)	**1:10 dilution**		**1:100 dilution**		**1:1000 dilution**	
	Found ($ng\ mL^{-1}$)	Recovery (%)	Found ($ng\ mL^{-1}$)	Recovery (%)	Found ($ng\ mL^{-1}$)	Recovery (%)
8.00	8.26±0.38	103	8.30±0.56	104	8.66±0.30	108
4.00	4.82±0.27	121	4.90±0.32	122	4.82±0.09	120
2.00	2.54±0.08	127	2.35±0.10	118	2.43±0.12	121
1.00	0.92±0.12	92	1.13±0.11	114	1.19±0.04	118
0.50	0.57±0.06	76	0.50±0.04	100	0.53±0.03	107
0.00	/	/	/	/	/	/

Format B						
Spiked ($ng\ mL^{-1}$)	**1:10 dilution**		**1:100 dilution**		**1:1000 dilution**	
	Found ($ng\ mL^{-1}$)	Recovery (%)	Found ($ng\ mL^{-1}$)	Recovery (%)	Found ($ng\ mL^{-1}$)	Recovery (%)
8.00	8.77±0.34	108	8.02±0.28	100	7.01±0.12	88
4.00	4.97±0.12	121	4.21±0.06	105	3.33±0.61	83
2.00	2.49±0.11	118	2.39±0.08	120	1.59±0.05	79
1.00	1.23±0.07	110	1.13±0.17	114	0.90±0.18	90
0.50	0.72±0.09	118	0.52±0.03	104	0.52±0.00	105
0.00	0.13±0.00	/	/	/	/	/

Format C						
Spiked ($ng\ mL^{-1}$)	**1:10 dilution**		**1:100 dilution**		**1:1000 dilution**	
	Found ($ng\ mL^{-1}$)	Recovery (%)	Found ($ng\ mL^{-1}$)	Recovery (%)	Found ($ng\ mL^{-1}$)	Recovery (%)
0.80	0.64±0.00	72	0.59±0.01	72	0.63±0.00	79
0.40	0.44±0.01	93	0.36±0.01	87	0.43±0.00	107
0.20	0.28±0.00	106	0.19±0.01	88	0.22±0.00	108
0.10	0.19±0.01	118	0.10±0.01	88	0.11±0.00	108
0.05	0.13±0.01	141	0.06±0.01	85	0.05±0.00	105
0.00	0.07±0.00	/	0.02±0.00	/	0.00±0.00	/
